# Transcriptional Derepression Uncovers Cryptic Higher-Order Genetic Interactions

**DOI:** 10.1371/journal.pgen.1005606

**Published:** 2015-10-20

**Authors:** Matthew B. Taylor, Ian M. Ehrenreich

**Affiliations:** Molecular and Computational Biology Section, Department of Biological Sciences, University of Southern California, Los Angeles, California, United States of America; University of Michigan, UNITED STATES

## Abstract

Disruption of certain genes can reveal cryptic genetic variants that do not typically show phenotypic effects. Because this phenomenon, which is referred to as ‘phenotypic capacitance’, is a potential source of trait variation and disease risk, it is important to understand how it arises at the genetic and molecular levels. Here, we use a cryptic colony morphology trait that segregates in a yeast cross to explore the mechanisms underlying phenotypic capacitance. We find that the colony trait is expressed when a mutation in *IRA2*, a negative regulator of the Ras pathway, co-occurs with specific combinations of cryptic variants in six genes. Four of these genes encode transcription factors that act downstream of the Ras pathway, indicating that the phenotype involves genetically complex changes in the transcriptional regulation of Ras targets. We provide evidence that the *IRA2* mutation reveals the phenotypic effects of the cryptic variants by disrupting the transcriptional silencing of one or more genes that contribute to the trait. Supporting this role for the *IRA2* mutation, deletion of *SFL1*, a repressor that acts downstream of the Ras pathway, also reveals the phenotype, largely due to the same cryptic variants that were detected in the *IRA2* mutant cross. Our results illustrate how higher-order genetic interactions among mutations and cryptic variants can result in phenotypic capacitance in specific genetic backgrounds, and suggests these interactions might reflect genetically complex changes in gene expression that are usually suppressed by negative regulation.

## Author Summary

Some genetic polymorphisms have phenotypic effects that are masked under most conditions, but can be revealed by mutations or environmental change. The genetic and molecular mechanisms that suppress and uncover these cryptic genetic variants are important to understand. Here, we show that a single mutation in a yeast cross causes a major phenotypic change through its genetic interactions with two specific combinations of cryptic variants in six genes. This result suggests that in some cases cryptic variants themselves play roles in revealing their own phenotypic effects through their genetic interactions with each other and the mutations that reveal them. We also demonstrate that most of the genes harboring cryptic variation in our system are transcription factors, a finding that supports an important role for perturbation of gene regulatory networks in the uncovering of cryptic variation. As a final part of our study, we interrogate how a mutation exposes combinations of cryptic variants and obtain evidence that it does so by disrupting the silencing of one or more genes that must be expressed for the cryptic variants to exert their effects. To prove this point, we delete the transcriptional repressor that mediates this silencing and demonstrate that this deletion reveals a similar set of cryptic variants to the ones that were discovered in the initial mutant background. These findings advance our understanding of the genetic and molecular mechanisms that reveal cryptic variation.

## Introduction

Cryptic genetic variants are standing polymorphisms that only exhibit phenotypic effects under atypical conditions, such as when specific genes are compromised or the environment dramatically changes [1–3]. Work in *Arabidopsis thaliana* (e.g., [4–6]), *Caenorhabditis elegans* (e.g., [7–9]), *Drosophila melanogaster* (e.g., [10–14]), multiple budding yeasts (e.g., [15–19]), and a number of non-model organisms (e.g., [20–26]) has shown that cryptic variation is abundant within and between species. Because it is so prevalent, cryptic variation could plausibly contribute to adaptation and phenotypic novelty [2, 27–29], as well as to disease susceptibility [30]. Yet due to their entirely conditional phenotypic effects, cryptic variants have proven difficult to study and are not understood as well as other classes of polymorphisms. In particular, the genetic and molecular mechanisms that suppress and uncover cryptic variation have yet to be fully determined.

For the purposes of this paper, we focus on the mechanisms by which functional disruption of specific ‘capacitor’ genes exposes the phenotypic effects of cryptic variants. This phenomenon is often referred to as ‘phenotypic capacitance’ or ‘evolutionary capacitance’, though for simplicity we refer to it as ‘capacitance’ [11, 31]. The first described capacitor was Hsp90, a chaperone that assists in the folding and stabilization of other proteins [11, 32]. Early research on capacitance suggested that Hsp90 might have distinct biochemical features that cause cryptic variation to be uncovered when it is compromised [4, 11, 32]. However, subsequent theoretical work showed that capacitance most likely occurs as a general consequence of gene regulatory network perturbation and that many genes might be able to act as capacitors [31]. Supporting this finding, a number of genes involved in chromatin regulation have also been shown to be capacitors of cryptic variation [15, 33, 34] and to even phenocopy the effects of Hsp90 perturbation [34].

**Fig 1:**
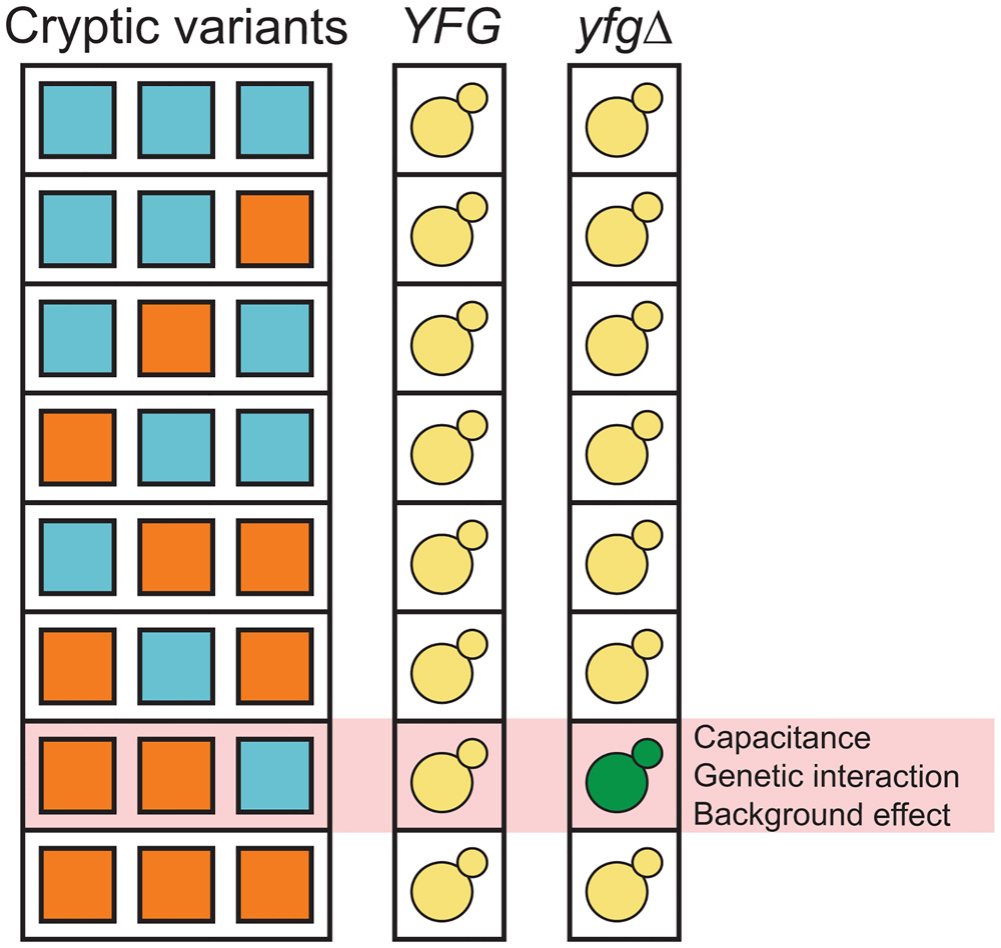
Capacitance, higher-order genetic interactions, and genetic background effects might be related phenomena that involve interactions among capacitating mutations and cryptic variants. ‘*YFG*’ and ‘*yfgΔ*’ refer to the wild type and mutant alleles of a gene that can genetically interact with cryptic variants. The green yeast indicates the combination of a capacitating mutation and cryptic variants that shows a phenotypic effect.

More recent work suggests that capacitance depends not only on the perturbation of capacitors but also on the specific cryptic variants that are present. This is because cryptic variants themselves can play an important role in capacitance by genetically interacting with and ‘potentiating’ the phenotypic effects of their capacitors [3, 17, 33, 35–37]. The genetic architecture of this potentiating cryptic variation has not been characterized in detail [38], but may involve complex epistatic interactions between multiple cryptic variants and capacitating mutations (i.e., higher-order genetic interactions) [39]. In such a scenario, the phenotypic effect of a given capacitating mutation would depend on the cryptic variants with which it co-occurs, with the mutation having an effect only in certain genetic backgrounds [40] (Fig 1). This possibility is not unfounded, as several recent studies suggest that genetic background effects can involve higher-order genetic interactions among *de novo* or induced mutations and sets of cryptic variants [41–43].

**Fig 2:**
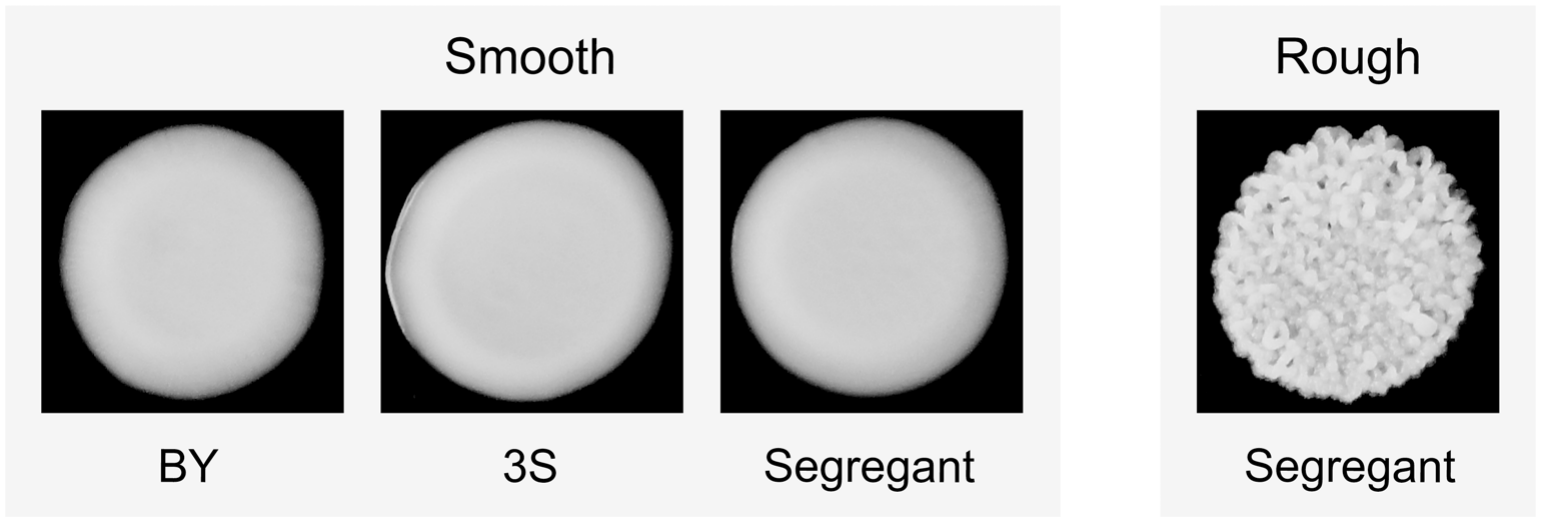
Colony morphology phenotypes that occur in the BYx3S cross in the presence of *ira2Δ*2933. BY, 3S, and most segregants show a smooth phenotype, while a small fraction of segregants show a rough phenotype.

We recently described an experimental system that can be used to study how higher-order genetic interactions among mutations and cryptic variants result in capacitance [42]. In our previous paper, we showed that a *de novo* mutation in *IRA2*, a negative regulator of the Ras-cAMP-PKA (Ras) pathway [44, 45], uncovers sets of interacting cryptic variants that influence colony morphology in *Saccharomyces cerevisiae*. This mutation (*ira2Δ*2933) occurred spontaneously while we were generating a cross of the lab strain BY4716 (‘BY’) and a derivative of the clinical isolate 322134S (‘3S’) [46, 47], and results in a truncated, partially functional Ira2 protein that lacks 117 amino acids relative to its wild type form. When the *ira2Δ*2933 lesion is present in specific haploid recombinants in the BYx3S cross, it causes a change in colony morphology from ‘smooth’ to ‘rough’ (Fig 2).

Through comprehensive genetic mapping experiments, we showed that *ira2Δ*2933 induces the rough phenotype when it co-occurs with specific combinations of cryptic variants at four or more genes [42]. To better understand these higher-order genetic interactions, we cloned all of the genes involved in one of the combinations. This resulted in the identification of two transcriptional activators that heterodimerize and function downstream of the Ras pathway (*FLO8* [48] and *MSS11* [49]), a structural protein that plays a role in vesicle formation (*END3* [50, 51]), and an enzyme that helps cells detoxify themselves of endogenous redox stress (*TRR1* [52]). Most of the rough individuals in our past study had the genotype *END3* ^BY^ *FLO8* ^3S^ *ira2*Δ2933 *MSS11* ^BY^ *TRR1* ^3S^. However, we also provided evidence for a more complex genotype involving *END3* ^3S^ that requires specific alleles at two additional loci.

In this paper, we complete our efforts to determine the genetic basis of *ira2Δ*2933-dependent rough morphology in the BYx3S cross under our standard assay conditions. We show that in addition to the previously identified five-way genetic interaction, a six-way interaction can also cause the trait. Specifically, individuals with the genotype *END3* ^3S^ *FLO8* ^3S^ *ira2*Δ2933 *MSS11* ^BY^ exhibit the rough phenotype if they possess BY alleles at two other transcription factors that are regulated by the Ras pathway [53, 54]: the activator *MGA1* [55] and the repressor *SFL1* [56, 57]. This suggests that the rough phenotype arises due to genetically complex changes in the regulation of Ras target genes. We examine the role of *ira2Δ*2933 in these regulatory changes and find that it alleviates the silencing of *FLO11*, a gene that encodes a cell surface protein required for rough morphology. We also show that this ability to disrupt *FLO11* repression is not unique to *IRA2*. These results illustrate how higher-order combinations of cryptic variants can confer the potential for capacitance to specific genetic backgrounds and indicate that capacitating mutations may reveal cryptic phenotypic potential by causing transcriptional derepression.

## Results

### *END3* ^3S^ and *ira2Δ*2933 are involved in a six-way genetic interaction

**Fig 3:**
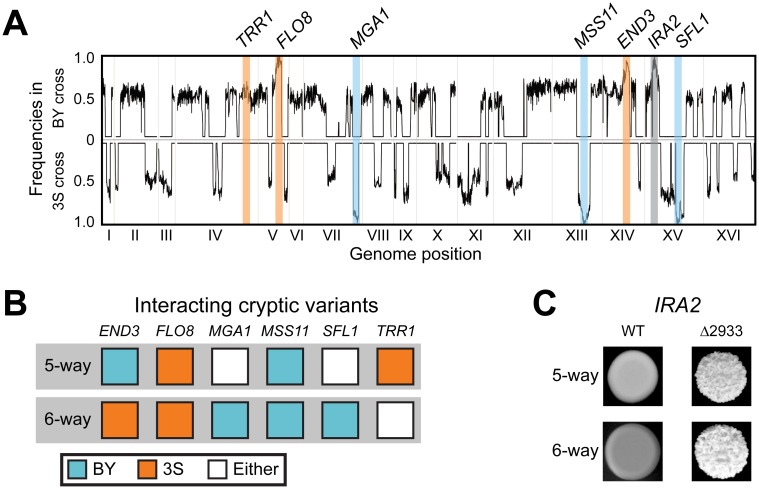
Characterization of the six-way genetic interaction. (**A**) Allele frequency plots for BY and 3S second iteration backcross populations of *END3* ^*3S*^ rough strains. Fixed loci are denoted with a blue, orange, or grey bars depending on whether the BY, 3S, or mutant alleles, respectively, were detected at a locus. The allele frequencies were estimated by averaging data in sliding windows containing 10 SNPs. (**B**) Cryptic variants involved in the five- and six-way interactions. (**C**) Dependence of both genetic interactions on the *ira2Δ*2933 mutation.

To determine the specific combination of alleles involved in rough morphology in an *END3* ^3S^ background, we generated new mapping populations by mating an *END3* ^3S^ rough segregant from a (BYx3S)x3S backcross to BY and 3S (Methods). Throughout the paper, the term ‘backcross’ refers specifically to these ((BYx3S)x3S)xBY and ((BYx3S)x3S)x3S matings. Because *END3* ^3S^ segregated in the BY backcross, we genotyped rough individuals recovered from this population to determine the allele of *END3* they carried (Methods). In total, we obtained 63 and 88 rough *END3* ^3S^ individuals from the BY and 3S backcrosses, respectively. We then pooled cells from these rough individuals by cross and performed bulk segregant mapping by sequencing [58, 59] (Methods). We found that the more complex genetic interaction involves a specific combination of alleles at six loci, with individual loci detected on Chromosomes V, VII, XIII, and XIV, and two loci identified on Chromosome XV (Fig 3A and 3B). The chromosome XIV locus corresponds to *END3* ^3S^, while allele replacements in a backcross segregant that carried the six-way interaction confirmed that *FLO8* ^3S^, *MSS11* ^BY^, and *ira2*Δ2933 underlie the Chromosome V, XIII, and XV-1 loci, respectively (Fig 3C and S1 Fig; Methods). The new mapping data also allowed us to delimit the Chromosome VII and XV-2 loci, which we were unable to clone in our prior study [42], to a single gene (*MGA1*) and five genes (*SFL1*, *ARP8*, *LSC1*, *SUF5*, *THI80*), respectively. We used allele swaps to show that the BY alleles of *MGA1* and *SFL1*, which respectively encode an activator and a repressor that are regulated by the Ras pathway, are the causal alleles at these loci (S1 Fig). These results show the six-way interaction occurs in individuals with the genotype *END3* ^3S^ *FLO8* ^3S^ *ira2*Δ2933 *MGA1* ^BY^ *MSS11* ^BY^ *SFL1* ^BY^ (Fig 3B). Thus, the differences between the five- and six-way interactions involve which *END3* allele is involved and whether specific alleles of *MGA1*, *SFL1*, and *TRR1* are required (Fig 3B).

### The two interactions fully account for rough morphology in the presence of *ira2Δ*2933

Based on our genetic mapping results in this paper and our past work [42], we have identified alleles of six genes (*END3*, *FLO8*, *MGA1*, *MSS11*, *SFL1*, *TRR1*) that genetically interact in two different combinations with *ira2Δ*2933 (Fig 3B and 3C). We tested whether these two allele combinations fully explain rough morphology in the BYx3S *ira2Δ*2933 cross by generating a new BYx3S cross in which 3S carried *ira2Δ*2933 (Methods). As our past work focused on matings of segregants to BY or 3S, this population enabled us to test for the first time the effects of all possible combinations of BY and 3S alleles in the presence of *ira2Δ*2933. Among 42 rough individuals that we recovered, 40 (95.2%) carried the five-way interaction, while two (4.8%) carried the six-way interaction. The five-way interaction should occur twice as often as the six-way interaction, yet the observed ratio was 20:1. This may be due to linkage between *END3* and a locus at which the BY allele confers a strong selective advantage during random spore isolation (see Figure S2B from [42]). Alternatively, the enrichment of rough individuals carrying the five-way interaction could simply have occurred because the sample of rough individuals in this experiment was small. Nevertheless, our observation that all the examined rough individuals harbored either the five- or six-way interactions suggests that we have completely determined the genetic basis of rough morphology in the BYx3S *ira2Δ*2933 cross under our experimental conditions.

### *FLO11* expression is needed for rough morphology

**Fig 4:**
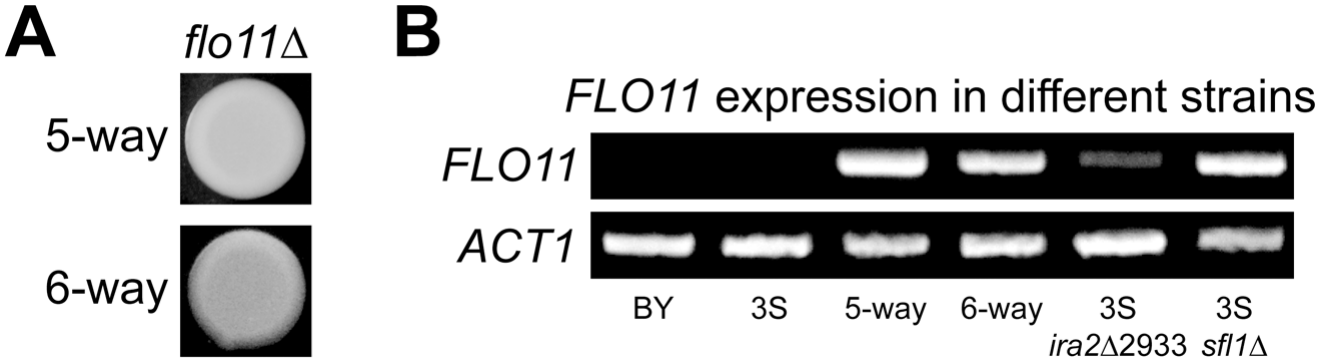
*FLO11* is required for rough morphology and shows differential expression across genetic backgrounds. **(A)** Deletion of *FLO11* leads to smooth morphology in both the five- and six-way genetic interaction backgrounds. **(B)** RT-PCR of *FLO11* and the housekeeping gene *ACT1* in multiple genetic backgrounds. *FLO11* is not expressed in BY or 3S, but is expressed in recombinants that carry the five- and six-way genetic interactions. *FLO11* is also expressed in 3S *ira2Δ*2933 and 3S *sfl1Δ* strains.

Rough morphology in the BYx3S cross likely arises due to genetically complex changes in the regulation of Ras target genes. Such a possibility is supported by the finding that four Ras-regulated transcription factors [54] harbor cryptic variants involved in the rough phenotype, as well as by the fact that these cryptic variants are revealed by a capacitating mutation in *IRA2*, a negative regulator of Ras signaling. A gene that is likely influenced by these genetic factors is *FLO11*, which encodes a cell surface glycoprotein that facilitates cell-cell adhesion and is thought to be regulated by Flo8-Mss11, Mga1, and Sfl1 [60, 61]. To determine if expression of the rough phenotype due to the five- and six-way interactions requires *FLO11*, we deleted the gene from a nearly isogenic line possessing the five-way interaction and a backcross segregant carrying the six-way interaction (Methods). This was sufficient to convert both of these strains from rough to smooth (Fig 4A), indicating that both genetic interactions are *FLO11*-dependent. RT-PCR showed that *FLO11* is expressed in individuals carrying the five- and six-way interactions, but not in BY or 3S (Fig 4B; Methods). These results suggest expression of the rough phenotype requires active transcription of *FLO11*.

### *ira2*Δ2933 and *SFL1* deletion cause *FLO11* expression in 3S

We tested whether *ira2Δ*2933 influences *FLO11* expression by introducing the lesion into BY and 3S, and conducting RT-PCR (Methods). Each strain remained smooth after this manipulation, which was expected because they both lack a complete set of alleles that can give rise to rough morphology. Furthermore, BY *ira2*Δ2933 did not express *FLO11*, likely because this strain carries a nonsense allele of *FLO8*, the major transcriptional activator of *FLO11* [62]. However, introduction of *ira2*Δ2933 into 3S, which possesses a functional allele of *FLO8*, converted *FLO11* from a silenced to an actively transcribed state (Fig 4B). Given that *ira2Δ*2933 alleviated repression of *FLO11* in 3S, we hypothesized that it might do so by indirectly inhibiting Sfl1, which is thought to negatively regulate *FLO11* and other targets of the Ras pathway when Ras signaling is low by recruiting the Ssn6-Tup1 corepressor complex [57], which in turn recruits the histone deacetylase Hda1 [63, 64]. To test this possibility, we deleted *SFL1* from 3S. This knockout phenocopied the results of introducing *ira2Δ*2933: 3S remained smooth, but expressed *FLO11* (Fig 4B). This suggests that *iraΔ*2933 disrupts Sfl1-mediated transcriptional repression of Ras target genes.

### Cryptic genetic variation uncovered by *SFL1* deletion

**Fig 5:**
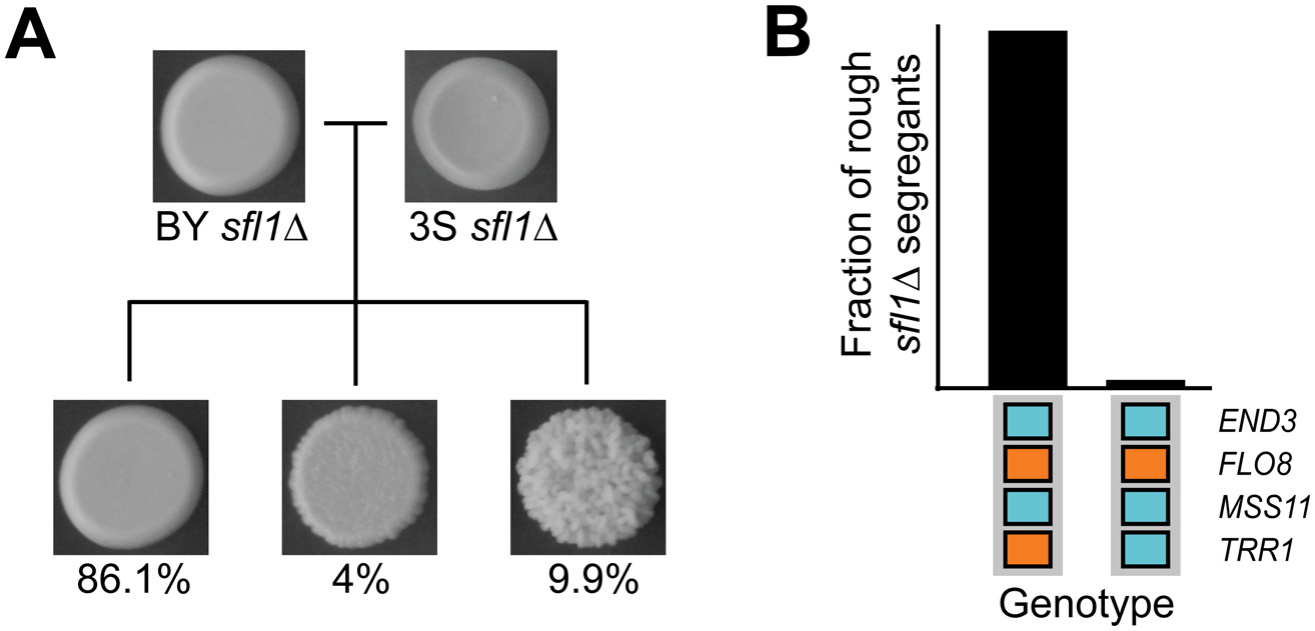
Deletion of *SFL1* reveals interacting cryptic variants. (**A**) Three phenotypic classes—smooth, bumpy, and rough—were observed among progeny from the BYx3S *sfl1Δ* cross. The proportion of segregants observed in each phenotypic class is shown below representative pictures for each class. (**B**) Genotypes observed among rough progeny from the BYx3S *sfl1Δ* cross.

To test whether loss of transcriptional repression by Sfl1 is sufficient to reveal the cryptic higher-order genetic interactions that specify rough morphology, we generated new BYx3S crosses. We first created a BYx3S cross that lacked the *IRA2* mutation and screened for rough morphology among thousands of recombinants (Methods). All segregants in this cross were smooth. We then constructed a cross in which BY and 3S carried wild type alleles of *IRA2*, but had *SFL1* deleted (Methods). Rough morphology, as well as a ‘bumpy’ intermediate phenotype that we previously reported (see Figure S4D and S1 Table in [42], as well as S1 Note), segregated in this *sfl1Δ* cross (Fig 5A). Genotyping of 44 rough *sfl1Δ* segregants showed that the rough phenotype is expressed in the *ira2Δ*2933 and *sfl1Δ* backgrounds largely due to the same cryptic variants (Methods). 43 (98%) of the rough *sfl1Δ* segregants possessed the genotype *END3* ^BY^ *FLO8* ^3S^ *MSS11* ^BY^ *TRR1* ^3S^, which also potentiates the five-way interaction involving *ira2Δ*2933 (Fig 5B). The other rough *sfl1Δ* segregant had the genotype *END3* ^BY^ *FLO8* ^3S^ *MSS11* ^BY^ *TRR1* ^BY^, which does not give rise to rough morphology in the presence of *ira2Δ*2933 (Fig 5B). None of the rough *sfl1Δ* segregants had a genotype resembling the six-way interaction involving *ira2Δ*2933. This could have occurred because *SFL1* ^BY^, which is required for the six-way interaction, is missing from the *sfl1Δ* cross; our sampling was biased due to the selectively advantageous locus that is linked to *END3*; or, as the detection of a rough *sfl1Δ* segregant with the *END3* ^BY^ *FLO8* ^3S^ *MSS11* ^BY^ *TRR1* ^BY^ genotype also suggests, *ira2*Δ2933 and *sfl1*Δ have similar but not identical molecular effects. Despite these differences between the *ira2Δ*2933 and *sfl1Δ* crosses, our results clearly show that transcriptional repression normally suppresses rough morphology and that multiple genes can act as capacitors by disrupting this negative regulation.

## Discussion

Across this manuscript and our previous paper [42], we have cloned six genes that harbor cryptic variants that interact in two specific allele combinations to determine the phenotypic effect of *ira2Δ*2933. These two genetic backgrounds can be viewed as potentiating genotypes that facilitate the expression of rough morphology in the presence of a capacitating mutation, such as *ira2Δ*2933. This finding is important because it shows sets of cryptic variants can genetically interact with each other and their capacitating mutation, and implies a conceptual link between capacitance, higher-order genetic interactions, and genetic background effects (Fig 1).

Given that four of the identified genes encode transcription factors, our work suggests complex gene regulatory changes underlie the expression of rough morphology in the BYx3S cross. This finding is consistent with theoretical results that have shown an important role for gene regulatory network perturbation in capacitance [31] and higher-order genetic interactions [65]. In our specific case, the role of *ira2Δ*2933 is likely to cause transcriptional derepression, which may enable the involved cryptic variants to collectively alter the gene regulatory network underlying colony morphology. Supporting such a role for derepression in the rough phenotype, we have shown that *IRA2* is not unique in its ability to act as a capacitor. Rather, *SFL1* can also serve as a capacitor of rough morphology, presumably because its deletion also causes transcriptional derepression.

Moving forward, fully understanding capacitance in the BYx3S colony morphology system will likely require defining the gene regulatory network underlying rough morphology and determining how it changes across combinations of cryptic variants and capacitating mutations. Such work can shed light on the individual and collective contributions of the identified cryptic variants to the rough phenotype; may reveal why *MGA1* ^BY^, *SFL1* ^BY^, and *TRR1* ^3S^ only have phenotypic effects in specific *END3* backgrounds; and might further clarify how multiple genes can act as capacitors of the same cryptic variants and trait. More generally, research along these lines has the potential to provide basic insights into how genetically complex, cryptic phenotypes are suppressed and uncovered.

Additionally, to our knowledge, the present study, when considered with [42], represents the first comprehensive genetic characterization of a genetic background effect in any organism. Our work demonstrates how genetic background effects can arise due to complex epistatic relationships between mutations and cryptic variants at multiple modifier loci, as others have previously suggested [43]. Our findings also indicate that multiple epistatic configurations of cryptic variants may enable a given mutation to show a phenotypic effect. Although these results advance understanding of the causes of genetic background effects, determining the generality of these findings will require dissecting other genetic background effects that involve different mutations, species, and traits.

## Materials and Methods

### Phenotyping of yeast colony morphology

All phenotyping experiments were performed on agar plates containing yeast extract and peptone (YP) with 2% ethanol as the carbon source (YPE). Prior to phenotyping, strains were grown to stationary phase in liquid YP with 2% dextrose (YPD). Cultures were manually pinned onto YPE and allowed to grow for five days at 30°C, and were then imaged using a standard digital camera.

### Generation of backcross segregants

Strains with opposite mating types were mixed together on a YPD plate and incubated for four hours at 30°C. A zygote from each cross was obtained by microdissection. To generate segregants, diploids were sporulated at room temperature using standard yeast sporulation procedures [66]. Once sporulation had completed, spore cultures were digested with β-glucuronidase and then plated onto YPE plates at a density of roughly 100 to 200 colonies per plate. Approximately 10 plates were screened per backcross.148 (BY backcross) and 88 (3S backcross) rough segregants were picked manually and streaked to obtain single cell isolates. The mating type of each of these strains was checked to confirm that they were indeed haploid. Segregants from the BY backcross could be either *END3* ^BY^ or *END3* ^3S^. In order to ensure sequenced strains possessed the *END3* ^3S^ allele, each segregant was genotyped using a nearby restriction marker (S1 Table). 63 of the 148 BY backcross progeny possessed the *END3* ^3S^ allele and were used for genetic mapping. We note that other multicellularity phenotypes (e.g., flocculation) segregated in the backcrosses, but were not strongly correlated with expression of the rough phenotype, implying they have different genetic architectures.

### Generation of *IRA2* wild type, *ira2Δ*2933, and *sfl1Δ* crosses

The BY and 3S strains used in the *ira2*Δ2933 and *sfl1*Δ crosses possessed the Synthetic Genetic Array marker system [67], which allowed for generation of large numbers of recombinant MAT**a** progeny. Regarding the *IRA2* wild type cross, we re-mated BY and 3S to produce a different diploid than the one used in [42]. For the *ira2*Δ2933 cross, the lesion was introduced into 3S using allele replacement techniques described below and then this 3S *ira2Δ*2933 strain was mated to a wild type BY strain. We designed the cross in this way because the *ira2*Δ2933 mutation originally occurred in the 3S allele of the gene. However, we note that we have never seen evidence for a genetic interaction between *ira2*Δ2933 and other genetic variants in *IRA2* ^3S^. As for the *sfl1*Δ cross, we constructed BY and 3S strains that lacked the entire coding region of *SFL1* using genetic engineering techniques described below. A BY/3S *sfl1Δ*/*sfl1Δ* diploid was then used to generate a population of BYx3S *sfl1Δ* recombinants. For each of the three crosses described in this section, diploids were generated and sporulated as described for the backcrosses, but sporulations were plated at low density onto YNB plates containing canavanine to select for haploid progeny. These were then replica plated on YPE to phenotype colony morphology. For each cross, around 20 plates containing roughly 100 to 200 colonies were screened.

### Bulk segregant mapping of rough morphology in the backcrosses

Each rough *END3* ^3S^ segregant from the backcrosses was grown to stationary phase as an individual, clonal culture. Cells from these stationary cultures were then mixed in equimolar fractions by backcross and DNA was extracted from the two pools using Qiagen G-tip columns. Whole genome sequencing libraries were prepared using the Illumina Nextera kit, with each of the backcross segregant pools barcoded with a unique sequence tag. The libraries were mixed together in equimolar fractions and sequenced on an Illumina MiSeq machine by the company Laragen, Inc. using 250 base pair (bp) x 250 bp reads. These sequencing reads were then mapped to the *S*. *cerevisiae* S288c reference and 322134S draft genomes (http://www.yeastgenome.org). S288c is the progenitor of BY, and to ensure high quality read mapping, reads from the BY and 3S backcrosses were mapped to S288c and 3S, respectively. Alignments were performed using the Burrows-Wheeler Aligner (BWA) version 7 with options mem -t 20 [68]. Based on these alignments, we obtained 73- and 122-fold genomic coverage, as determined by the average per site coverages, from the BY and 3S backcross populations, respectively. A custom Python script was used to assess genome-wide allele frequencies at 36,756 high confidence SNPs that had previously been identified by mapping Illumina sequencing reads for 3S to the S288c genome [42] (S2 Note; S2 Table). Loci influencing colony morphology were called as regions enriched at 95% frequency or higher when the data were averaged within running windows of 10 SNPs (S2 Note). Intervals containing causal genes were identified in the R statistical programming environment as the smallest regions that had mean allele frequencies above a threshold of 95% (S3 Note). Subsequent restriction typing experiments focused on individual segregants and the selected loci (see S1 Table) showed that the detected loci were in fact fixed, and that deviations from fixation occurred due to the presence of a small number of sequencing or read mapping errors. We note that Illumina data used for genetic mapping are available through the NCBI Sequence Read Archive under the study accession number SRP062432, as well as the sample accession numbers SAMN03956543 (BY backcross) and SAMN03956544 (3S backcross).

### Genetic engineering experiments

To generate allele replacement strains for *ARP8*, *LSC1*, *MGA1*, *SFL1*, *SUF5*, and *THI80*, a backcross segregant that expressed rough morphology due to the six-way genetic interaction was transformed using a modified form of adaptamer-mediated allele replacement [69]. Also, adaptamer-mediated allele replacement was used to introduce the *ira2Δ*2933 lesion into 3S. Transformations were conducted with two partially overlapping PCR products—a full-length amplicon of the gene of interest that was tailed at the 3’ end with the 5’ portion of the *kanMX* cassette and a copy of the *kanMX* cassette that was tailed on the 3’ end with part of the intergenic region downstream of the gene (as shown in Figure S1 of [70]). Knock-ins were identified using selection on G418 and verified by Sanger sequencing. Deletions were constructed using the CORE cassette [71]. Homology tails matching the 60 bases immediately up- and downstream of each gene were attached to the CORE cassette through PCR and introduced into cells using the Lithium Acetate method [72]. Selection for G418 resistance was used to screen for integration of the CORE cassette; correct integration was then checked using PCR. *SFL1* was deleted from BY and 3S, while *FLO11* was deleted from a nearly isogenic line and a backcross segregant harboring the five- and six-way genetic interactions, respectively. All primers used for genetic engineering are provided in S1 Table.

### Genotyping of causal alleles in *ira2Δ*2933 and *sfl1Δ* crosses

Markers within *END3*, *FLO8*, *MGA1*, *MSS11*, *SFL1*, and *TRR1* were genotyped using PCR and restriction digestion (S1 Table). These markers were identified from among the 36,756 high confidence SNPs that differentiate BY and 3S.

### RT-PCRs

Strains were grown to stationary phase in liquid YPD media at 30°C and pinned on to YPE agar plates. After four days of growth at 30°C, total RNA was extracted with the Qiagen RNeasy kit. cDNA was then generated with Superscript reverse transcriptase from Life Technologies. *ACT1*, a well-known housekeeping gene, was used as a control for our *FLO11* RT-PCRs. Strains that were used in the RT-PCR experiments are described in the main text. The specific primers that we used were taken from [73] and are provided in S1 Table.

## Supporting Information

S1 Fig Allele replacement results for *FLO8* ^BY^, *MGA1* ^3S^, *MSS11* ^3S^, and *SFL1* ^3S^ in the six-way genetic interaction. The role of *END3* ^3S^ was verified in [42], while the effect of *ira2*Δ2933 in this background is shown in Fig 2C.
(TIF)

S1 Table Primers used throughout the paper. (XLSX)

S2 Table SNPs used for genetic mapping. Coordinates are provided relative to the S288c reference genome.
(TXT)

S1 Note More information on the bumpy phenotype. (PDF)

S2 Note Python script for obtaining allele frequency data from an mpileup file. (TXT)

S3 Note R code for conducting the genetic mapping performed in this study. (TXT)
