# Supplementary figures and images for: Transcriptional Derepression Uncovers Cryptic Higher-Order Genetic Interactions
Source: PLoS Genet. 2015 Oct 20;11(10):e1005606. doi: 10.1371/journal.pgen.1005606 (PMC4618523; doi:10.1371/journal.pgen.1005606)

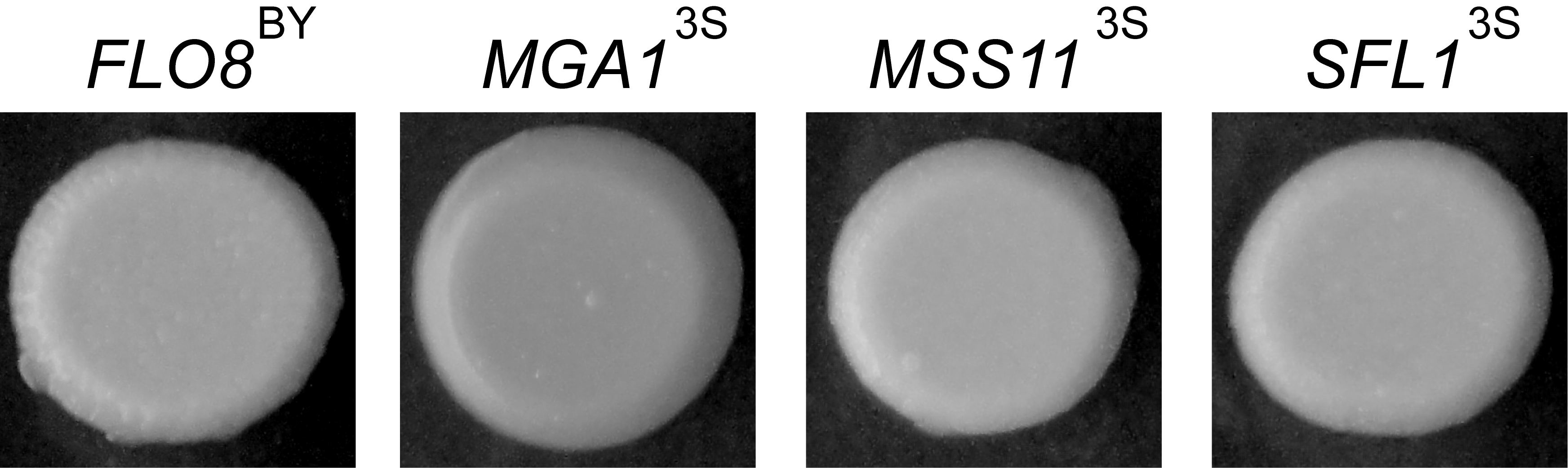

Supplement: S1 Fig — The role of END3 3S was verified in [42], while the effect of ira2Δ2933 in this background is shown in Fig 2C. (TIF) [file pgen.1005606.s001.tif]
