# Supplementary material for: Transcriptional Derepression Uncovers Cryptic Higher-Order Genetic Interactions
Source: PLoS Genet. 2015 Oct 20;11(10):e1005606. doi: 10.1371/journal.pgen.1005606 (PMC4618523; doi:10.1371/journal.pgen.1005606)
Supplement: S1 Note — (PDF) [file pgen.1005606.s004.pdf]

Note S1. More information on the bumpy phenotype.

We previously showed that some BYx3S segregants with the genotype *END3*^{BY} *FLO8*^{3S} *ira2*Δ2933 *MSS11*^{BY} can exhibit a bumpy phenotype that is visibly intermediate to the smooth and rough phenotypes (see Figure S4D and Table S1 in [1]). *TRR1* segregates among individuals with the bumpy phenotype (see Table S1 in [1]). This suggests that *TRR1* might not contribute to the bumpy trait and that other cryptic variants in the cross might be required for expression of the bumpy phenotype. In the current paper, we found that the bumpy phenotype also segregates in the *sfl1*Δ cross. We note that the bumpy phenotype typically occurs at a frequency of approximately 2 to 5% among BYx3S *ira2*Δ2933 segregants. Thus, the 4% frequency of bumpy individuals that we observe among BYx3S *sfl1*Δ segregants is comparable to that found among *ira2*Δ2933 individuals.

1. Taylor MB, Ehrenreich IM. Genetic interactions involving five or more genes contribute to a complex trait in yeast. PLoS genetics. 2014;10(5):e1004324.
